# The mechanism of high-mobility group box-1 protein and its bidirectional regulation in tumors

**DOI:** 10.17305/bb.2023.9760

**Published:** 2024-06-01

**Authors:** Zhongjia Tian, Lin Zhu, Yutong Xie, Huan Hu, Qunli Ren, Jianguo Liu, Qian Wang

**Affiliations:** 1 The Affiliated Stomatological Hospital of Zunyi Medical University, Zunyi, China; 2 Oral Disease Research Key Laboratory of Guizhou Tertiary Institution, School of Stomatology, Zunyi Medical University, Zunyi, China

**Keywords:** High-mobility group box-1 protein (HMGB1), tumor, autophagy, immunity, drug resistance

## Abstract

High-mobility group box-1 protein (HMGB1) is a nonhistone chromatin-related protein widely found in eukaryotic cells. It is involved in the transcription, replication, and repair of DNA to maintain nuclear homeostasis. It participates in cell growth, differentiation, and signal transduction. Recent studies showed that HMGB1 has a bidirectional regulatory effect on tumors by regulating TLR4/MYD88/NF-κB and RAGE/AMPK/mTOR signaling pathways. On the one hand, it is highly expressed in a variety of tumors, promoting tumor proliferation and invasion, while on the other hand, it induces autophagy and apoptosis of tumor cells and stimulates tumor-infiltrating lymphocytes to produce an anti-tumor immune response. At present, HMGB1 could be used as a target to regulate the drug resistance and prognostication in cancer. Clinical applications of HMGB1 in cancer need further in-depth studies.

## Introduction

The high-mobility group box-1 protein (HMGB1) is a highly conserved chromosomal protein within the high-mobility group protein family [1]. Intriguingly, it has the capacity to both promote tumor growth and stimulate anti-tumor immune responses [2]. HMGB1 plays a role in the development of oral [3], lung [4], colon [5], and breast cancer [6]. It exerts its bidirectional effects by binding to different types of receptors on various cells, thereby regulating numerous signaling pathways, such as NF-κB, ERK, and AMPK/mTOR [7, 8].

Autophagy is closely associated with drug resistance in malignant tumors, and HMGB1 can regulate the survival of tumor cells by inducing autophagy [9, 10]. This process could lead to an increase in drug resistance in a variety of malignancies, such as head and neck cancer [11], multiple myeloma (MM) [12], pancreatic cancer [13], and lung cancer [14]. Therefore, HMGB1 might serve as a regulatory target for drug resistance and a prognostic marker for malignant tumors, it has a promising clinical application as a target for tumor therapy and various HMGB1-targeted drugs have been developed recently [2]. At present, the search for new markers of tumor metastasis and recurrence holds significant clinical importance for diagnosis and targeted therapy. Tumor immunotherapy has evolved into one of the most effective treatments, with the role of HMGB1 in anti-tumor immunity also becoming a focal point of research. According to the latest studies on HMGB1, this review introduces recent advances in its bidirectional regulation and mechanism in tumors.

## The biological characteristics of HMGB1

### The structure of HMGB1

The *HMGB1* gene is located on chromosome 13 (13q12) and encodes 215 amino acids. HMGB1 protein includes two homologous DNA-binding regions and a short acidic C-terminus. The DNA-binding region comprises an A box (residues 9–79) and a B box (residues 95–163), while its C-terminus, formed by glutamic and aspartic residues (residues 186–215), contains binding sites for the Toll-like receptor (TLR) and the receptor for advanced glycation end products (RAGE) [15]. According to NMR and X-ray diffraction analysis, 80% of the amino acid sequences within these domains exhibit an α-helical conformation. However, the diversity at key sites leads to different biological functions: the A box can recognize and bind to AT-rich DNA fragments, while the B box can regulate the target DNA and affect cell proliferation by bending and altering its structure. The C-terminus of HMGB1 serves as the binding site to the specific receptor; it has been reported that the region spanning amino acids 201–205 of the C-terminus is involved in an antibacterial effect [16, 17].

## HMGB1 receptors

The primary receptors for HMGB1 are RAGE and TLRs [18, 19]. The interaction between HMGB1 and TLR4 can mediate signal transduction of the inflammatory response via MYD88, which activates the NF-κB signaling pathway, leading to the release of inflammatory factors, and ultimately triggering tumor antigen-specific T-cell immunity [20]. The binding of HMGB1 to RAGE can activate *Ras* oncogene, which in turn leads to the activation of the NF-kB inflammatory pathway mediated by the MAP kinase [18]. In addition, HMGB1 can interact with various molecules, such as LPS, IL-1β, CD24, and nucleosomes, eventually forming an inflammatory complex with these receptors and amplifying its own activity [21].

## Bidirectional roles of HMGB1

The distribution of HMGB1 in different cellular compartments leads to significant differences in biological function [22]. Under normal physiological conditions, HMGB1 is involved in various biological processes, such as DNA transcription, replication, and repair in the nucleus. However, under the influence of radio-chemotherapy or hypoxia, HMGB1 can be transferred to the extracellular environment through the active secretion by immunoreactive cells or the passive release by apoptotic necrotic cells. As a multifunctional protein, it acts as both a tumor promoter and an anti-tumor factor [23]. Remarkably, the two release ways of HMGB1 significantly differ in their molecular modifications, active release is induced by the hyperacetylation of the nuclear localization sequences (NLSs) following post-translational modification, while passive release manifests through the different redox states of HMGB1 [15].

Once HMGB1 is released into the extracellular environment, it activates damage-associated molecular patterns (DAMPs). Thereafter, HMGB1 can act as a cytokine or chemokine, binding to cell surface receptors, such as TLRs and RAGE, prompting immune cells to secrete various inflammatory cytokines, creating a microenvironment in favor of tumor initiation and progression [18, 24, 25]. However, radiotherapy or chemotherapy could cause immunogenic tumor cell death (ICD). ICD is an apoptotic cell death marked by the surface translocation of calreticulin and the release of ATP and HMGB1, among other factors. During ICD, antigen-presenting cells are activated and subsequently phagocytosed by dendritic cells. When the immunogenic signal reaches a certain threshold, anti-tumor immune responses are triggered [16].

## Bidirectional regulations of HMGB1 in tumor autophagy

**Figure 1.**
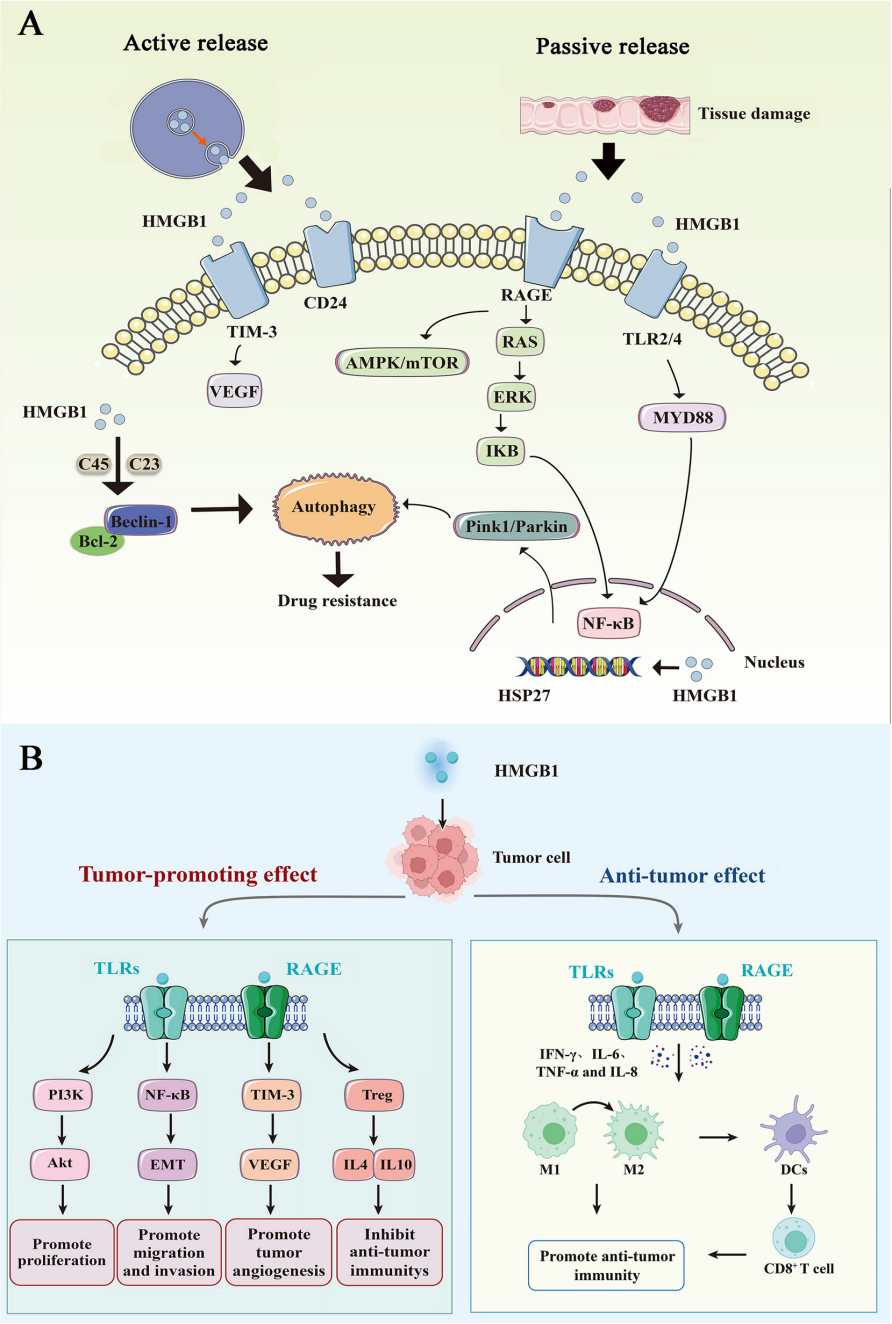
**Schematic diagram of the regulatory mechanism of HMGB1.** (A) Active and passive release of HMGB1. HMGB1 mediated autophagy: In the nucleus, HMGB1 could upregulate the transcription of HSP27 by the Pink1/Parkin pathway and induce autophagy. In the cytoplasm, HMGB1 could interact with Beclin-1 and lead to autophagy. HMGB1 could also bind to the RAGE receptor, activate Ras oncogene, and activate autophagy by ERK and AMPK/mTOR pathways. HMGB1-mediated inflammation: The combination of HMGB1 and TLR2/4 could mediate the signal transduction of inflammatory reactions through MYD88, activate NF-κB signal pathway, and lead to the release of inflammatory factors. (B) Bidirectional regulation mechanism of HMGB1 in tumor. Tumor-promoting effect: HMGB1 could promote the proliferation, tumor angiogenesis, invasion, and metastasis of tumor cells through the PI3K/AKT/NF-κB/VEGF/EMT signaling pathway, thus modulating Treg that in return inhibit anti-tumor immunity. Anti-tumor effect: HMGB1 combined with TLR4 promotes maturation of DC, upregulates the ratio of M1/M2 in tumor microenvironment, increases recruitment of tumor-specific CD8+ T cells, and mediates anti-tumor immune response. HMGB1: High mobility group box-1; RAGE: Receptor for advanced glycation end products; TLR: Toll-like receptor.

HMGB1 serves as a vital regulator of autophagy in tumor cells, inducing autophagy through multiple avenues. The Pink1/Parkin pathway plays a key role in maintaining mitochondrial health [26]. In the nucleus, HMGB1 can upregulate the transcription of HSP27 via the Pink1/Parkin pathway and induce autophagy. In the cytoplasm, HMGB1 at C23 and C45 interacts with Beclin-1, mediates the ERK/MAPK pathway, and promotes the phosphorylation of Bcl-2, which ultimately induces the segregation of the Beclin-1/Bcl-2 complex, leading to autophagy [27]. Outside the cells, HMGB1 can bind to the RAGE receptor and activate autophagy via ERK and AMPK/mTOR pathways [8]. Autophagy regulated by HMGB1 has both positive and negative effects as it can promote tumor cell proliferation as well as induce tumor cell death [28, 29] (Figure 1A).

## Tumor-promoting effects of HMGB1

### HMGB1 promotes tumor cell proliferation

In a study investigating oral squamous cell carcinoma (OSCC), it was found that extracellular HMGB1 could be overexpressed as a cancer cell growth factor and upregulate the expression of RANKL in osteoclasts by activating RAGE and TLR4 receptors. Autocrine HMGB1 can promote the proliferation of OSCC cells and facilitate osteoclast formation and bone destruction. Therefore, inhibiting HMGB1 and its downstream signaling pathways may be a mechanism-based antitumor approach for treating advanced oral cancer [3]. Advanced HNSCC can infiltrate and metastasize to bone, causing HNSCC-associated bone pain (HNSCC-BP), leading to dysphagia, which severely compromises the quality of patients’ lives. The primary cause is the active release of HMGB1 by HNSCC cells into the bone marrow’s extracellular space, which, in turn, promotes osteoclast formation. This subsequently results in bone resorption with the effect of an electrogenic proton pump, causing an inward flow of calcium ions in the neuronal axis and bone pain. Therefore, targeting HMGB1 may provide an effective therapy [30].

In gastric cancer research, it was found that extracellular HMGB1 could be overexpressed as a cancer cell growth factor. Upon binding to the RAGE receptor, it activates the MAPK or PI3K/AKT signal pathway, leads to the phosphorylation of ERK1/2 and promotes the proliferation of gastric cancer cells [31]. It is worth noting that autophagy can also influence the release of HMGB1. Increased HMGB1 release following autophagy induction in gastric cancer cells activates ERK, MAPK, AKT, and JNK signal pathways, thereby promoting cell proliferation [28].

Feng and Chen [32] reported that silencing of HMGB1 combined with docetaxel could inhibit the proliferation of prostate cancer cells, promote cell apoptosis, and hinder tumor formation. HMGB1 also plays a key role in the onset and development of pancreatic cancer. Previous studies suggested that among various cancer types, pancreatic cancer cells release the highest levels of HMGB1 when compared with breast and lung cancers [33]. Moreover, exogenous HMGB1 can activate p-GSK 3β through the Wnt signaling pathway, upregulate the expression of Bcl-2 and Ki67, and downregulate the expression of E-CA in pancreatic cancer cells, thus fostering the proliferation of pancreatic cancer cells [34].

### HMGB1 promotes tumor cell migration and invasion

The erythropoietin-producing hepatocyte (EPH) receptor, the largest subfamily within the receptor tyrosine kinase family, is involved in physiological processes, such as embryonic development, angiogenesis, and axon guidance [35]. EPHB4, one of the typical EPH members, is highly expressed in OSCC tissues. It has the capacity to enhance the proliferation and metastasis of OSCC by activating the HMGB1-mediated NF-κB signaling pathway, ultimately accelerating the malignant progression of cancer [36]. Furthermore, the diverse single-nucleotide polymorphisms (SNPs) of HMGB1 are associated with OSCC, and the 1177GG genotype of HMGB1 is correlated with lymphatic metastasis and tumor stage in OSCC, suggesting that HMGB1 haplotypes may be relevant to the susceptibility to oral cancer [37].

Epithelial–mesenchymal transition (EMT) is a crucial process in the metastasis of tumor cells. Increasing evidence showed that the invasiveness and migration capability of tumor cells in the initial stages of the metastatic cascade are enhanced by EMT [38]. In non-small cell lung cancer, HMGB1 activates the TLR4/NF-κB pathway and upregulates the expression of MMP2, Twist, and MMP9, thus accelerating EMT and fostering migration and invasion [39].

### HMGB1 promotes tumor angiogenesis

Tumor vasculature formation is closely linked to cancer progression; under a hypoxic tumor microenvironment, angiogenic factors are transferred from tumor cells to endothelial cells, triggering proliferation, migration, and vasculature formation within the tumor [40]. TIM-3 serves as a significant regulator of autoimmune and cancer immune responses [41]. When combined with TIM-3, HMGB1 stimulates the production of vascular endothelial growth factor (VEGF) and induces vascularization [42]. Kam et al. [43] discovered that HMGB1 could modulate the tumor microenvironment and promote tumor vascularization by regulating B cells in esophageal squamous carcinoma. Moreover, the overexpression of HMGB1 could aid the proliferation and migration of B cells, and transcriptional analysis also revealed a high abundance of angiogenic genes is high in the migratory B cells. Similarly, the interaction of HMGB1 and RAGE could induce the expression of VEGF by activating the NF-κB pathway in OSCC [44].

### HMGB1 inhibits anti-tumor immunity

Regulatory T cells (Treg) play a crucial role in the maintenance of immune tolerance [45]. In cases of HNSCC, HMGB1 can be overexpressed in tumor cells of HNSCC, and its high levels can act as chemoattractants for Treg. This facilitates the survival of Treg and augments its suppressive capacity in a dose-dependent manner, thereby modulating body immunity [11]. A comprehensive analysis of serum cytokines and immune mediators in HNSCC patients revealed a spike in HMGB1 levels during cancer recurrence, which was accompanied by high levels of IL-4 and IL-10. These findings suggest that one of the potential mechanisms in cancer recurrence could be the secretion of HMGB1 and the release of inhibitory cytokines by immune cells, which, in turn, adjust the tumor immune microenvironment [46].

HMGB1 can regulate the tumor micro-environment of OSCC by mediating the NF-κB signaling pathway. It has been confirmed in various OSCC cell lines (CAL-27/SCC25/SCC9) that HMGB1, upon binding to TLR4, activates the NF-κB pathway, regulates macrophage polarization by upregulating the expression of IL-10 and TGF-β, and enhances the expression of PD-L1, thus promoting tumor immune escape [36, 47]. Furthermore, the same condition was observed in HSC-3 and FaDu HNSCC cell lines.

### HMGB1 maintains the energy metabolism of tumor cells

HMGB1 is closely linked to tumor energy metabolism. In the case of pancreatic cancer, Kang et al. [48] discovered that HMGB1 elevates the levels of RAGE within mitochondria, thereby amplifying the activity of mitochondrial complex I and the production of ATP in tumor cells. This, in turn, facilitates the metabolism and proliferation of tumor cells. Glutamine, being the substrate source of the TCA cycle and a regulator of redox homeostasis, plays an important role in the adaptation and survival of tumor cells. HMGB1 is capable of promoting glutamine metabolism, activating the mTORC2/AKT/C-MYC positive feedback circuit, modulating the expression of glutamine synthetase (GS), and inducing mTORC1 signal transduction to diminish the expression of SIRT4 on glutamate dehydrogenase (GDH) [49]. Additionally, it fosters the malignant proliferation and self-renewal of hepatocellular carcinoma cells, while obstructing immunotherapy through PD-L1+ exosome activity [50].

## Anti-tumor effects of HMGB1

### HMGB1 maintains the activity of autophagy

The translocation of HMGB1 to the cytoplasm has been proven to be an inducement of autophagy in many cancers. HMGB1 can interact with Beclin-1 in the cytoplasm of radioresistant OSCC cells and promote the formation of the Beclin-1 and PI3KIII complex, thus upregulating autophagy flux. Moreover, the HMGB1-Beclin-1 complex in the cytoplasm can also mitigate chemotherapy-induced oxidative stress and play a protective role in oxidative stress-mediated apoptosis by regulating autophagy [51].

During tumor development, intracellular HMGB1 acts as an antitumor protein to maintain genomic stability and autophagic activity [27]. HSPB1 is a type of heat shock protein with cytoprotective effects, such as maintaining protein homeostasis, stabilizing the structure of the cytoskeleton, and enhancing the stress response of cells [52]. In vitro studies demonstrated HMGB1’s role in the autophagic clearance of dysfunctional mitochondria, mediating mitochondrial function by regulating the expression of HSPB1. Under a stress state, HMGB1 defends against mitochondrial abnormalities and improves the effect of autophagic clearance. Accordingly, in the absence of HMGB1 or HSPB1, there is significant mitochondrial disruption along with dysfunction of aerobic respiration, suggesting that HMGB1 and HSPB1 could coordinate autophagy following mitochondrial damage [53, 54].

### HMGB1 promotes anti-tumor immunity

TLR agonists can upregulate the ratio of M1/M2 in the tumor microenvironment, enhancing the antigen presentation of TAMs and the differentiation of tumor-specific T cells. Local application of TLR agonists also augments the recruitment of tumor-specific CD8+T cells in the tumor and draining lymph nodes [55]. Similarly, in HPV-negative OSCC patients receiving radiotherapy, their poor prognosis was potentially associated with the infiltration of CD68+ macrophages, where HMGB1 may serve as a target to inhibit macrophage recruitment and improve therapeutic efficacy [56].

Thermochemotherapy can induce the expression of DAMPs and enhance immunogenicity in OSCC cells. Extracellular HMGB1 transmits a “danger” signal to the immune system, binding to TLR4 on DCs and activating the TLR4-mediated signaling pathway. This promotes the maturation of DCs and antigen processing and presentation, thereby mediating the anti-tumor immune response and inhibiting the growth of distal metastases [57].

Moreover, HMGB1, in conjugation with TNF-α participates in the anti-tumor immune response of M1 macrophages in breast cancer [39]. Extracellular HMGB1 can induce M1-like polarization of TAMs in the tumor microenvironment of glioblastoma, activating the ERK1/2/NF-κB/NLRP3 inflammatory pathway and promoting the release of various cytokines (e.g., IFN-γ, IL-6, TNF-α, and IL-8) [58]. These results also suggest that the combination of immunotherapy and radiotherapy may improve the efficacy of tumor therapy.

## HMGB1 in tumor therapy

### HMGB1 and its targeted medicine

The presence of HMGB1 in serum has been significantly correlated with poor prognosis in laryngeal squamous cell carcinoma. Survival analysis demonstrated that high expression of HMGB1 is a crucial clinicopathological parameter, indicating a decreasing trend in 5-year survival rates [59]. Similarly, the dynamic changes of HMGB1 during radiotherapy are correlated with the prognosis of HNSCC patients. Elevated levels of HMGB1 in the serum of HNSCC patients reflect ICD levels and are related to multiple confounding factors, such as inflammation and infection. Hence, HMGB1 holds promise as a biomarker to monitor tumor response during the chemoradiotherapy of HNSCC [60]. Evodiamine, a novel anti-tumor drug, can inhibit proliferation and induce apoptosis of tumor cells; in an OSCC transplantation tumor rat model, it could target the binding of HMGB1 and RAGE, thus modulating the AGE/RAGE downstream signaling system, thereby inhibiting the proliferation, invasion, and angiogenesis in OSCC [44].

Sunitinib is a widely used tumor angiogenesis inhibitor, but common side effects include cardiomyocyte apoptosis and cardiac dysfunction. Xu et al. [61] reported that the cardiomyocyte apoptosis induced by sunitinib could be significantly inhibited in the absence of HMGB1. To enhance treatment safety, a specific inhibitor of HMGB1, glycyrrhizin, was combined with sunitinib to reduce cardiotoxicity. Additionally, glycyrrhizin could modulate the HMGB1/RAGE and HMGB1/TLR4 signaling pathways in melanoma, disrupting tumor metastasis, and growth [62].

Neutrophil extracellular trap networks (NETs), which are reticulated structures composed of DNA histones and proteins released by activated neutrophils, govern the intrinsic immune response mediated by neutrophils and regulate tumor progression and metastasis [63]. Currently, HMGB1 has been confirmed as a promoting molecule for NETs. Chen et al. [64] found that metformin can inhibit the formation of HMGB1-induced NETs, thus reducing cancer aggressiveness.

Numerous studies have established that many natural active ingredients regulate disease development through HMGB1. For instance, ginsenoside can improve the inflammatory microenvironment of cardiomyocytes and enhance the efficiency of the feedforward loop by modulating the HMGB1/NF-κB signaling pathway [65]; in acute kidney injury, quercetin can reduce cell injury and apoptosis by inhibiting HIF-1α in the lncRNA NEAT1/HMGB1 signaling pathway [66]. Furthermore, molecular docking analysis revealed that potential active ingredients of Dao-Chi powder can inhibit inflammatory responses and oxidative stress by regulating the Nrf2/HO-1/HMGB1 signaling pathway [67]. These studies indicate that HMGB1 could be targeted by many active ingredients. Recently, network pharmacology utilized high-throughput screening, network visualization, and network analysis to reveal the complex network relationship between drugs, targets, and diseases [68]. The combination of network pharmacology and natural active components will aid in exploring the mechanism and the detailed function of HMGB1 in cancer treatment.

### HMGB1 and drug resistance

LncRNA H19, recognized as a significant oncofetal gene, has garnered considerable attention. It plays a crucial regulatory role in the migration of cancer cells by targeting its downstream genes. Its function in drug resistance has emerged as a hot research topic; in laryngeal cancer, upregulated H19 is associated with autophagy-induced drug resistance. In addition, H19 can regulate the expression of HMGB1 by targeting miR-107, demonstrating an inhibitory effect on autophagy both in vitro and in vivo, and enhance the sensitivity of laryngeal squamous cell carcinoma to cisplatin [69].

Dexamethasone has the capability to inhibit the growth of MM, but long-term use may lead to drug resistance or even relapse. Currently, there is growing research focused on the impact of autophagy on drug resistance. DEPTOR, an endogenous mTOR inhibitor, has been verified to induce drug resistance in MM through autophagy [70]. To study the drug resistance of HMGB1 in MM, Guo et al. [12] employed dexamethasone in the treatment of *HMGB1* gene knockout myeloma and discovered that the apoptosis of MM cells induced by dexamethasone could be promoted when the expression of HMGB1 is inhibited. In contrast, in the control group without the treatment of dexamethasone, the phosphorylation levels of Akt and p70S6k increased, and the levels of LC3A/B-II decreased significantly. These findings suggest that dexamethasone can inhibit autophagy following *HMGB1* knockout, and HMGB1 might impact the resistance of MM and regulate autophagy through the DEPTOR/mTOR/Akt pathway.

**Table 1:** The function of HMGB1 and its interaction factors in different types of malignant tumors

**Cancer type**	**Biological process/Molecular function**	**Key factors/Pathways**	**References**
*Tumor-promoting effects*			
Oral squamous cell carcinoma	Promotes proliferation; Promotes migration and invasion	RAGE and TLR4; NF-κB signaling pathway	[3, 36, 44]
Head and neck cancer	Promotes formation of osteoclasts; Inhibits anti-tumor immunity	Treg, IL-4 and IL-10	[30, 43]
Gastric cancer	Promotes proliferation	MAPK or PI3K/AKT signaling pathway	[28, 31]
Pancreatic cancer	Promotes proliferation	p-GSK 3β and Wnt signaling pathway	[33, 34]
Non-small cell lung cancer	Promotes migration and invasion	TLR4/NF-κB signaling pathway, MMP2, Twist, and MMP9	[39]
Esophageal squamous carcinoma	Promotes tumor vascularization; Increases ATP production	NF-κB signaling pathway	[43, 47]
Liver cancer	Promotes malignant proliferation	mTORC2/AKT/C-MYC	[50]
*Anti-tumor effects*			
Oral squamous cell carcinoma	Maintains the activity of autophagy; Mediates the anti-tumor immune response	Beclin-1 and PI3KIII; TLR4/DCs	[50, 56]
HPV-negative OSCC	Promotes anti-tumor immunity	CD8+T cells	[55]
Breast cancer	Promotes anti-tumor immunity	TLR4, TNF-α	[6]
Glioblastoma	Activates anti-tumor immune responses	ERK1/2/NF-κB/NLRP3 signaling pathway, IFN-γ, IL-1β, IL-6, CCL2, TNF-α, and IL-8	[57]
Multiple myeloma	Induces apoptosis	LC3A/B-II, DEPTOR/mTOR/AKT signaling pathway	[12]
Liver cancer	Promotes autophagy	MAPK/mTOR	[14]

RAGE: Receptor for advanced glycation end products; OSCC: Oral squamous cell carcinoma; HMGB1: High-mobility group box-1 protein; TLR: Toll-like receptor; Treg: Regulatory T cells; AKT: Protein kinase B; MAPK: Mitogen-activated protein kinase; NF-κB: Nuclear factor kappa B; ERK1/2: Extracellular signal-regulated kinase 1/2; IFN-ɣ: Interferon gamma; IL: Interleukin; TNF-α: Tumor necrosis factor alpha; NLRP3: Nucleotide oligomerization domain (NOD)-like receptors (NLRs) 3; MMP: Matrix metalloproteinase; PI3K: Phosphoinositide 3-kinase; p-GSK 3β: Phospho-glycogen synthase kinase 3 beta; Wnt: Wingless-related integration site; mTORC2: Mammalian target of rapamycin complex; DCs: Dendritic cells; CCL2: Chemokine (C-C motif) ligand 2; LC3A/B-II: Microtubule-associated protein 1 light chain 3 alpha/beta-2; DEPTOR: Disheveled, Egl-10, and Pleckstrin (DEP) domain-containing mTOR-interacting protein; mTOR: Mammalian target of rapamycin.

Doxorubicin (DOX) is a traditional chemotherapeutic drug and a standard component in the treatment of advanced liver cancer [71]. However, tumor resistance restricts the effectiveness of DOX [72]. Li et al. [14] found that as the DOX dose in liver cancer increased, the expression of extracellular HMGB1 gradually elevated, activating the AMPK/mTOR pathway and facilitating autophagy; however, autophagy regulated by HMGB1 may result in the inhibition of DOX-induced apoptosis and an increase in drug resistance [73].

In short, drug resistance is closely tied to tumor autophagy. One of the primary driving forces is the increased autophagic flux of tumor cells prompted by the high expression of extracellular HMGB1. Correspondingly, when HMGB1 is knocked out, the sensitivity of tumor cells to chemotherapy can be enhanced, leading to chemotherapy-induced apoptosis. HMGB1 may serve as a potential therapeutic target in improving the efficacy of chemotherapeutic drugs and regulating drug resistance by affecting autophagy.

## Conclusion

HMGB1 plays a key role in the development of various tumors by regulating the PI3K/AKT/NF-κB/VEGF/EMT and AMPK/mTOR signaling pathways, and it can act as both a tumor suppressor and an oncogenic factor (Table 1 and Figure 1B). In the nucleus, HMGB1 binds with DNA to adjust the assembly of proteins with specific DNA targets; besides transcription regulation, HMGB1 also plays multiple roles in tumor development as an extracellular signaling protein, such as regulating proliferation and invasion, angiogenesis, autophagy, and apoptosis of tumor cells, participating in tumor immune response, and maintaining the metabolic growth of tumor cells, among others. Due to the bidirectional regulation of HMGB1 on the tumor, it can promote the recruitment of macrophages during chronic release, and subsequently alter the tumor microenvironment to induce malignant transformation, and promote tumor cell proliferation, migration, and invasion. Conversely, during acute mass release, HMGB1 and its receptor TLR4 interact to stimulate dendritic cell activation and induce ICD, thus inhibiting carcinogenesis and eliciting a specific immune response. Based on these findings, when tumors are subjected to immunotherapy, consideration ought to be given to the appropriate level of HMGB1, controlling for its release type and guiding its affinity to dendritic cells as a new treatment approach.

HMGB1-regulated autophagy can promote not only tumor cell proliferation but also tumor cell apoptosis. Disrupting the relationship between HMGB1 and Beclin-1/Bcl-2 may be an effective way to regulate autophagy, thus inhibiting the survival of cancer cells. Drug resistance is a primary cause of chemotherapy failure as it has been proven that HMGB1-mediated autophagy is closely related to drug resistance of tumors. Knocking out HMGB1 could reverse the drug resistance of tumors and increase their sensitivity to chemotherapy. In addition, HMGB1 is also the target of many natural active components in a variety of tumors.

In summary, HMGB1 is not only a biomarker for prognostic analysis and immunotherapy response but also a potential target for tumor therapy. Therefore, further study on its bidirectional regulatory roles and mechanisms in tumors is necessary to provide effective chemotherapy options and drug design for the clinical treatment of malignant tumors.
